# An Evaluation of Coverage and Compliance of Mass Drug Administration 2006 for Elimination of Lymphatic Filariasis in Endemic Areas of Gujarat

**DOI:** 10.4103/0970-0218.39242

**Published:** 2008-01

**Authors:** Pradeep Kumar, PB Prajapati, Deepak Saxena, Abhay B Kavishwar, George Kurian

**Affiliations:** Department of Community Medicine, Government Medical College, Surat - 395 001, India; 1 Department of NVBDCP, Gandhinagar - 382 010, Gujarat, India

**Keywords:** Lymphatic filariasis, mass drug administration

## Abstract

**Background:**

Mass drug administration (MDA) means once-in-a-year administration of diethyl carbamazine (DEC) tablet to all people (excluding children under 2 years, pregnant women and severely ill persons) in identified endemic areas. It aims at cessation of transmission of lymphatic filariasis.

**Objective:**

What has been the coverage and compliance of MDA in Gujarat during the campaign in December 2006?

**Study Design:**

Cross-sectional population based house-to-house visit.

**Setting:**

Urban and rural areas in Gujarat identified as endemic for filariasis where MDA 2006 was undertaken.

**Study Variables:**

*Exploratory* - Rural and urban districts; *Outcome* - coverage, compliance, actual coverage, side effects.

**Analysis:**

Percentage and proportions.

**Results:**

Twenty-six clusters, each comprising 32 households from six endemic districts, yielded an eligible population of 4164. The coverage rate was 85.2% with variation across different areas. The compliance with drug ingestion was 89% with a gap of 11% to be targeted by intensive IEC. The effective coverage (75.8%) was much below the target (85%). Side effects of DEC were minimum, transient and drug-specific. Overall coverage was marginally better in rural areas. The causes of poor coverage and compliance have been discussed and relevant suggestions have been made.

## Introduction

Lymphatic filariasis (LF) is an important public health problem next to Malaria in India.(1) WHO had recently called on member states to identify the global elimination of LF as a public health priority.(2) The International Task Force for Disease Eradication too had identified LF as one of the seven infectious diseases considered eradicable or potentially eradicable.(3) Several interventions have been tried in recent times to deal with this health problem. Mass drug administration (MDA), which means once-in-a-year administration of diethyl carbamazine (DEC) tablet to all people (excluding children under 2 years, pregnant women and severely ill persons) in identified endemic areas, is one of them.(4) It aims at cessation of transmission of LF in the community. MDA in combination with other techniques has already eliminated LF from Japan, Taiwan, South Korea and Solomon Islands and markedly reduced the transmission in China.(5,6) MDA has been as effective as 12-day therapy, as a public health measure, with lesser side effects, thus enhancing public compliance and decreasing delivery costs.(7) It does not require complex management and infrastructure, and can be integrated into the existing primary healthcare (PHC) system.(4) MDA is already in place in 32 of the 83 endemic countries.(8) In India, MDA with single dose of DEC (6 mg/kg body weight) was taken up as a pilot project covering 41 million population in 1996-97 and was extended to 77 million population by 2002.(4) In order to achieve the elimination of LF by 2015 under the National Health Policy, National Filarial Day (NFD) was proposed to be observed every year starting from 2004 in the endemic districts.(9) Based on microfilaria surveys and the line listing of lymphedema cases, Gujarat had identified eight districts and one town as endemic for the disease, and accordingly they were included for observing MDA since 2004. A detailed report covering all the aspects of MDA (pre, during and post) evaluation with recommendations and suggestions has been submitted to the state government.(10) The present communication deals only with the evaluation of coverage (distribution of drug to the community) and compliance (actual drug consumption) of MDA in December 2006 in endemic areas of Gujarat.

## Materials and Methods

MDA was undertaken in identified endemic areas in December 2006. For operational reasons, it could not be conducted in the districts of Jamnagar and Junagarh; therefore, the activities took place in six districts (Surat, Valsad, Navsari, Amreli, Rajkot and Porbandar) and one town (Dabhoi in Vadodara). The activities under MDA involved administration of DEC tablets to eligible population from endemic area by health staff and Integrated Child Development Scheme (ICDS) functionaries referred as drug distributors (DD) make house-to-house visits on select dates in December 2006. DEC was administered to all people (excluding children under 2 years, pregnant women and severely ill persons) with the instruction to ingest the tablet preferably on the spot. Evaluation of the MDA was done by PSM faculties of medical colleges in Gujarat. The specific objectives dealt in this communication were:

1. To find out the coverage, compliance, coverage-compliance gap (CCG) and actual coverage rates.
2. To report the side effects of DEC, which may be either drug-specific or due to microfilaria in blood (mild fever, headache or giddiness).

### Selection of the survey area

Four clusters per district (one from urban and three from rural areas) for six districts and two clusters for Dabhoi were selected for the survey. Thus, a total of 26 clusters were studied. The survey was done 1 week after the MDA, and the coverage reported by the health system was used to select the clusters. Selection was done as per the following criteria:

1. One PHC with >80% coverage.
2. One PHC with 50-80% coverage.
3. One PHC with up to 50% coverage.
4. One ward from urban area with highest coverage in Dabhoi and two wards with highest and lowest reported coverage were selected.

In case if there was no urban area involved, two PHCs were selected with coverage between 50 and 80%. In addition, if there was no PHC falling in a particular coverage category, the PHC was selected from neighboring category.

A total of 32 households (HHs) in each cluster were selected in such a way that the entire ward/village was represented. For this purpose, the area was divided into four quadrants, and in each quadrant, a central point was identified and the first house was selected randomly (any number between 1 and 9) and thereafter another seven HHs (total eight) serially (open with available family members) were covered. The exercise was repeated in other three quadrants. In fact this was an improvement over 30 HHs suggested per cluster by NVBDCP for evaluation.(4)

All the data were collected in a pre-designed and structured proforma. After data collection, analysis was done with the help of Epi Info. Various rates for eligible population, coverage, compliance, CCG and effective coverage (product of coverage and compliance) were calculated for the entire state. Rates were also calculated separately for different districts, Dabhoi town and rural and urban areas. Because the evaluation was carried out in a small sample so to get the estimates for entire population covered, 95% confidence intervals for effective coverage were also calculated.(11)

## Results

A total of 26 clusters (four per district and two for Dabhoi) including eight from urban and 18 from rural areas were studied. Together, these 26 clusters covered a total of 835 HHs (579 - rural and 256 - urban) and yielded a population of 4288 (2926 - rural and 1362 - urban). In the studied clusters, against a population of 4288, 4164 (97.1%) were eligible for MDA [Table 1]. Adhering to the criteria of NVBDCP,(4) the eligible population in various districts and town varied between 96.2 and 98.3%. The proportions of eligible population in rural and urban clusters were 96.8 and 97.7%, respectively. The rest was either below 2 years of age (98), pregnant females (22) or severely ill (4). Out of 4164 persons eligible, 3546 (85.2%) received DEC [Table 1]. Against overall coverage rate of 85.2%, it was highest in Porbandar (99.6%) and lowest in Navsari (77.3%). The remaining (*n* = 618) although eligible did not get the drug for various reasons. The common reasons were DD did not visit (32.2%), followed by DD did not give drug (23.3%), houses were locked or people were not available (19.4%) and nonreceipt of drug due to misclassification (elderly 11.7%, children 1.2% and sick 9.6%). Twelve persons (1.2%) refused to accept the drug. The reason of “nonvisit by DD” was seen more in Rajkot, Valsad and Navsari districts. Nonadministration of DEC without any reason was more in Rajkot. Similarly, the misclassification of persons as elderly or young or severely ill was seen more in Navsari and Valsad districts.

**Table 1:** Distribution of population of surveyed districts

District	Total population	Eligible population		Population covered (out of eligible)	
			
		*n*	%	*n*	%
Amreli	682	659	96.6	592	89.8
Navsari	609	595	97.7	460	77.3
Porbandar	531	513	96.6	511	99.6
Rajkot	733	709	96.7	502	70.8
Surat	654	630	96.3	562	89.2
Valsad	708	695	98.2	570	82.0
Dabhoi	371	363	97.8	349	96.1
Total	4288	4164	97.1	3546	85.2

Compliance refers to the actual consumption of drug by the community. Our survey was necessary to ascertain the compliance in people other than those who consumed the drug “on the spot”. Similarly, the gap between the coverage and compliance identifies an area of intervention by motivating people to consume the drug (compliance) made available to them by the health system (coverage) [Table 2]. Compliance rate (ingestion of drug by those who received it) was 89% with lowest in Navsari (72.4%) and highest in Porbandar (99.0%). CCG as a whole was 11%. Area-wise, it was very high in Navsari (27.6%) and lowest in Porbandar (1%), whereas at rest of the places it was between 7 and 14% [Table 2]. A total of 390 persons accounted for this gap. The main reason for this was refusal without any reason (63.3%). Some of them (19.5%) did not take it for fear of side effects and the rest (17.2%) either forgot to take or misplaced the drug. Effective coverage rate is the end product of coverage by the health system and compliance by community. It was less than the target (85%) for the entire state as a whole. It was highest in Porbandar (98%) and lowest in Navsari (56%) [Table 2]. In fact, except the district of Porbandar and Dabhoi town, no district could achieve the targeted coverage of 85%. Even with optimism, we take into account the upper limits of 95% confidence intervals; the coverage in the districts of Rajkot, Navsari and Valsad was less than 85%.

**Table 2:** Compliance rate, coverage-compliance gap and effective coverage rate

District	Eligible population	DEC given by DD	Consumed (compliance rate)		CCG (%)	Effective coverage rate (%)	95% Confidence intervals
						
			*n*	%			
Amreli	659	592	526	88.9	79.8	11.1	73.3-86.9
Dabhoi	363	349	324	92.8	89.3	7.2	80.1-99.6
Navsari	595	460	333	72.4	56.0	27.6	50.3-63.3
Porbandar	513	511	506	99.0	98.6	1.0	90.4-100.0 (107.5)
Rajkot	709	502	431	85.9	60.8	14.1	55.3-66.8
Surat	630	562	505	89.9	80.2	10.1	73.5-87.5
Valsad	695	570	531	93.2	76.4	6.8	70.2-83.2
Total	4164	3546	3156	89.0	75.8	11.0	73.2-78.5

CCG - Coverage-compliance gap, DEC - Diethyl carbamazine

Both coverage and compliance were marginally better in rural areas than in urban areas, and accordingly the actual coverage too was better in rural areas. However, it was less than 85% in both rural and urban areas [Table 3].

**Table 3:** Drug coverage and compliance rates in urban and rural settings

Area	Coverage rate (%)	Compliance rate (%)	CCG (%)	Effective coverage rate (%)	95% confidence intervals
Rural (*n* = 2833)	85.5	90.6	9.4	76.2	73.1-79.5
Urban (*n* = 1331)	84.4	87.8	12.2	74.2	69.7-78.9
Total (*n* = 4164)	85.2	89.0	11.0	75.8	73.2-78.5

CCG - Coverage-compliance gap

### DEC consumption and side effects

Out of 3156 persons who consumed DEC, only 68 (2.15%) reported some side effects. The most common side effect was nausea and/or vomiting (24) followed by sedation/drowsiness (20), headache (11), vertigo (8), body pain (3) and acidity (2). All these side effects developed following the consumption of DEC and were drug-induced. They appeared within hours of drug intake and disappeared within 2-3 days. In none of the cases, any treatment was taken. We did not come across the allergic type of side effects (fever, local inflammation, pruritus, etc.) anywhere. Although more clusters and population were surveyed in rural area, the side effects were reported more from urban areas (70%) than from rural areas (30%).

## Discussion

A high coverage (>85%) in endemic areas, which is sustained for 5 years, is required to achieve the interruption of transmission and elimination of disease in India.(4) The major challenge with the currently available drugs is to attain this high coverage. Current approaches to drug delivery are able to achieve only 40-60% coverage if MDA is executed by regular health services.(12) There is an urgent need for more effective drug delivery strategies that are adapted to regional differences and variations in health sector development.(13) A special challenge exists for drug delivery in urban settings, while other problems are the low priority given to LF and poor compliance with DEC treatment. All these problems require powerful advocacy tools and strategies.(14) The 85.2% coverage observed by us was unsatisfactory because under the MDA, the target was to ensure effective coverage of 85% - a product of coverage as well as compliance. The main reasons for noncoverage were that workers could not cover the population or did not administer drug and importantly the misclassification of persons rendering them not eligible. It can be improved by making efficient microplans, improved supervision and emphasizing more strongly the selection criteria in training.

CCG helps to understand why people fail to consume the drug. It was around 11% and needs to be bridged with side by side efforts through IEC from all possible channels to motivate people for ingestion (preferably on the spot) of the drug. Same emphasis has been made elsewhere as well.(4,7) Except in 17.6% cases (fear of side effects), reasons for noncompliance were trivial, such as forgot to take, misplaced the drug or “no reason”. It seems that LF is not perceived as a serious public health problem or people think that they will not be affected by this disease. All these point out to one thing that there is no resistance in the community for DEC; however, more important is to emphasize on supervised “on the spot” DEC consumption. One reason commonly given by the community for not consuming DEC on the spot was that it causes gastric upsets and so they prefer to take it after the meal. In this regard, a suggestion came to us that DD may carry small packets of biscuits (costing Rs. 2) to facilitate spot consumption of DEC.

Effective coverage rate is the end product of coverage of the health system and compliance by community. In fact this should be 85% or above for the elimination of disease.(4) Overall, it was 75.8% for the entire state and further being low in districts like Navsari. Here, both coverage and compliance have been good. Good coverage in the absence of good compliance and similarly motivated community (for good compliance) with poor coverage will be of little use.

As such, the side effects were very few and they were also minor, transient and drug-specific. However, they also need to be addressed as they constitute the cause of noncompliance. Information about the Rapid Response Team (RRT) must be widely publicized in order to increase the faith of people and will indirectly result in better compliance. Although more population was surveyed in rural population, the side effects were reported more from urban areas, which may be due to the high awareness and likelihood of relating the symptoms with the drug intake. As a whole, the situation in terms of coverage and compliance was marginally better in rural areas, which can be attributed to the availability of infrastructure in these areas.

## Conclusions and Recommendations

1. Effective coverage, after taking into account the coverage and compliance, was almost same in both rural and urban areas (76%), but was less than the targeted coverage (85%) under the program.
2. Coverage and compliance were marginally better in rural areas. Coverage-compliance gap too was around 10% in both areas. There was hardly any resistance in the community for the program and only 1.2% of those who were given the drug refused to accept it. Similarly, refusal to taking drug for fear of side effects accounted for less than 20% of noncompliance. Efforts are needed to reduce this gap before increasing the coverage. It needs motivating and sensitizing the community through IEC.
3. Incidence of side effects after MDA was minimal. All side effects were mild and needed no medical intervention; however, the community was largely unaware of RRT.
4. DD hardly insisted on supervised “on the spot” administration of drugs; therefore, supervised drug intake was nil or poor and the commonest answer was “will take after meal”. Efforts should be made to insist on “on the spot” consumption. This alone can bring down the coverage-compliance gap considerably.
5. Inclusion criteria were misunderstood. In some districts (Navsari, Valsad and Amreli), DD, by mistake of their own, did not give DEC to anyone whose age was more than 50 or 60 years. Similarly, DEC was not given to persons who were having diabetes or hypertension. In our evaluation, we considered such persons eligible. Therefore, the coverage and effective coverage decreased in our evaluation. Training of DD in future should focus on the point that anybody who is above 2 years of age, nonpregnant and not critically ill (having some acute illness or hospitalization) must receive the drug.
6. Various modes of pre-MDA IEC can be utilized such as radio, TV, cable, newspapers, recorded messages or SMS (mobile or landline phones) and should be done just few days before the campaign. IEC should focus on the following:
  - Threat perception of filariasis was very poor among people as it is not a visible disease, but still it is a threat as many people are at risk, and taking DEC only once in a year can prevent it.
  - The single-dose DEC once in a year is an effective preventive tool while in treatment a person may have to take it for 21 days. Even many practicing doctors are also not clear about it.
7. The MDA program in one place clashed with another governmental program (Chintan Shivir). It affected the supervision of MDA. In another district, there was a delay from the office of CDPO in issuing the orders to AWW; therefore, the activities were also delayed. Priority given to IPPI by the authorities also affected the working of MDA 2006. Efforts are to be made to avoid clashing of such programs.
